# Muscle allele-specific expression QTLs may affect meat quality traits in *Bos indicus*

**DOI:** 10.1038/s41598-021-86782-2

**Published:** 2021-04-01

**Authors:** Jennifer Jessica Bruscadin, Marcela Maria de Souza, Karina Santos de Oliveira, Marina Ibelli Pereira Rocha, Juliana Afonso, Tainã Figueiredo Cardoso, Adhemar Zerlotini, Luiz Lehmann Coutinho, Simone Cristina Méo Niciura, Luciana Correia de Almeida Regitano

**Affiliations:** 1 Post-Graduation Program of Evolutionary Genetics and Molecular Biology, Center of Biological Sciences and Health, Federal University of São Carlos, São Carlos, SP Brazil; 2 Post-Doctoral Fellow, Department of Animal Science, Iowa State University, Ames, IA USA; 3 Embrapa Pecuária Sudeste, P. O. Box 339, São Carlos, SP 13564-230 Brazil; 4 Embrapa Informática Agropecuária, Campinas, SP Brazil; 5 Department of Animal Science, University of São Paulo/ESALQ, Piracicaba, SP Brazil

**Keywords:** Genetics, Animal breeding, Functional genomics, Gene expression, Gene regulation, Genetic markers, Genome, Genomics, Genotype, Animal biotechnology

## Abstract

Single nucleotide polymorphisms (SNPs) located in transcript sequences showing allele-specific expression (ASE SNPs) were previously identified in the *Longissimus thoracis* muscle of a Nelore (*Bos indicus*) population consisting of 190 steers. Given that the allele-specific expression pattern may result from *cis*-regulatory SNPs, called allele-specific expression quantitative trait loci (aseQTLs), in this study, we searched for aseQTLs in a window of 1 Mb upstream and downstream from each ASE SNP. After this initial analysis, aiming to investigate variants with a potential regulatory role, we further screened our aseQTL data for sequence similarity with transcription factor binding sites and microRNA (miRNA) binding sites. These aseQTLs were overlapped with methylation data from reduced representation bisulfite sequencing (RRBS) obtained from 12 animals of the same population. We identified 1134 aseQTLs associated with 126 different ASE SNPs. For 215 aseQTLs, one allele potentially affected the affinity of a muscle-expressed transcription factor to its binding site. 162 aseQTLs were predicted to affect 149 miRNA binding sites, from which 114 miRNAs were expressed in muscle. Also, 16 aseQTLs were methylated in our population. Integration of aseQTL with GWAS data revealed enrichment for traits such as meat tenderness, ribeye area, and intramuscular fat . To our knowledge, this is the first report of aseQTLs identification in bovine muscle. Our findings indicate that various *cis*-regulatory and epigenetic mechanisms can affect multiple variants to modulate the allelic expression. Some of the potential regulatory variants described here were associated with the expression pattern of genes related to interesting phenotypes for livestock. Thus, these variants might be useful for the comprehension of the genetic control of these phenotypes.

## Introduction

Allele-specific expression (ASE) or allelic expression imbalance is a pattern that reflects the expression difference between copies of a gene from each chromosome (i.e., alleles)^1^. When ASE is parentally guided, it is named genomic imprinting, caused by epigenetic modifications with *cis* action, which result in the monoallelic expression of genes essential for mammal growth and development^2^.

Genes showing ASE in their transcripts, as evidenced by the analysis of single nucleotide polymorphisms (SNP) alleles within them, are called ASE genes. ASE genes have been shown to affect phenotypes for livestock, such as adipogenesis and lipid metabolism in pigs^3^. The knowledge about regions with ASE and their regulatory mechanisms is relevant to improve animal breeding programs by increasing the accuracy of predictive models^4^. De Souza et al.^5^ identified SNPs in transcribed regions showing allele-specific expression patterns (ASE SNPs) in the muscle of Nelore (*Bos indicus*) related to meat tenderness, an important trait for meat consumer’s approval^6,7^. Many of these SNPs were located within genes responsible for essential biological functions for muscle development and meat tenderness^5^.

Recent studies have explored putative regulatory mechanisms that may be responsible for ASE and genomic imprinting^8–10^. For instance, ASE can be caused by differential methylation in regulatory regions of the two parental alleles^11^. DNA methylation is a well-known imprinting mark in CpG islands^12^, DNA regions with clusters of cytosine followed by guanine dinucleotides. This type of epigenetic modification, when presented in promoters, can suppress transcription when the CpG islands are hypermethylated, while active transcription shows no methylation or few isolated methylation events^11^. Another mechanism involved with the ASE pattern is the presence of differences on microRNA (miRNA) binding sites between the alleles^13,14^, once miRNAs can impair the translation by degrading the target mRNA sequence^15^. Similarly, sequences showing an affinity for a transcription factor (TF), i.e., a transcription factor binding site (TFBS), may promote or suppress the transcription of the allele in phase with the given TFBS. Thus, in a heterozygous locus, if the regulatory region of only one allele has an affinity with the transcription factor (TF), this *cis*-regulatory variant can also lead to ASE^16^.

To discover regulatory mechanisms causing ASE, we can execute an initial screening analysis to identify SNPs associated with a given transcript's unequal allele expression pattern: the ASE-quantitative trait loci (aseQTLs)^17^. From this initial screening, aseQTLs that present any evidence of being related to interesting phenotypes and regulatory mechanisms affecting allelic expression can be prioritized for further analysis. AseQTLs can contribute to the understanding of expression regulation of genes associated with economic traits and how alleles under selection are expected to be expressed in the offspring^18–23^.

Herein, we investigated aseQTLs associated with allelic expression imbalance, evidenced by the number of reads corresponding to different SNP alleles in transcripts, previously described in a Nelore experimental population^5^. We then analyzed its involvement with predicted regulatory mechanisms, such as DNA methylation, whether they contained TFBS or miRNA binding sites. This study aimed to add a new knowledge layer about genomic regulation in Nelore muscle, describing potential mechanisms involved in the allelic expression of genes related to relevant phenotypes in bovine.

## Results

### aseQTLs analysis

**Figure 1:**
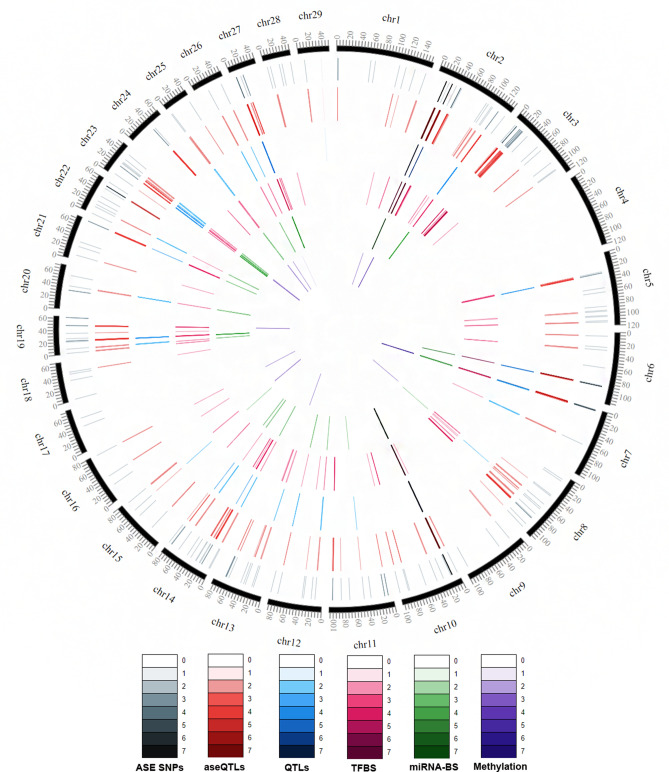
SNPs and aseQTLs distribution through the bovine chromosomes, overlap with previous QTL studies and predicted aseQTL regulatory mechanisms (TFBS, miRNA binding site, or methylated site). ASE SNPs: SNPs in transcripts with allele-specific expression, only the ASE SNPs with significant aseQTLs are displayed. aseQTL: identified allele-specific quantitative trait loci (aseQTLs). Overlapped: aseQTLs that overlaps with previous GWAS data made from our group. TFBS: aseQTLs that possibly enable the TF binding in only one allele. miRNA-BS: aseQTLs that potentially modify miRNA binding sites in only one allele. Methylated: methylated aseQTLs. The color intensity increases with increasing SNPs density in the plot scale (1 bp windows). This figure was made using the software CircosVCF^82^.

We retrieved a total of 82,384 SNPs after filtering the original genotype file (429,513 SNPs from 190 Nelore samples), considering a 1 Mb sized window around the 820 SNPs with significant ASE. Despite this initial dataset, only 22,470 tests were valid due to the requirement of 10 heterozygous samples for both the aseQTL candidate and the SNP marking allelic imbalance. In this context, only 192 SNPs with ASE were tested in the aseQTL analysis. Allelic imbalance ratio values were compared between groups of homozygous and heterozygous animals for each SNP to be tested as aseQTL. AseQTL analysis resulted in 1134 significant aseQTLs (*P* value ≤ 0.05), associated with 126 SNPs marking allelic imbalance of 85 genes. AseQTLs reference alleles have a mean frequency of 0.67, ranging from 0.49 to 0.94. Figure 1 represents ASE SNPs and aseQTLs distributions through the chromosomes.

Three SNPs marking ASE were also identified as aseQTLs: rs136209194, located in the intergenic region, which acts like an aseQTL for the rs132817153 SNP; rs109842586 (*XIRP2*), associated with the ASE pattern of the rs109372848 SNP; and rs136097891 (*MTUS1*), an aseQTL for the rs109005284 SNP *(MTUS1).* AseQTLs association results can be found in Supplementary Table 1.

The average number of aseQTLs per ASE SNP was nine (ranging from one to 56). The rs134422650 marker was associated with the highest number of aseQTLs (56). The second one was the rs110850310, located in the *HSPA1A* gene, with 38 aseQTLs. Figure 1 shows that some genomic locations had several aseQTLs (red ring) because they had multiple SNPs with allelic imbalance (grey ring) nearby, for example, between 15 and 20 kb positions of the chromosome 2 or between 10 and 15 kb of the chromosome 10.

Most aseQTLs affected the ASE marked by a unique SNP. Still, we found the rs132798564 aseQTL simultaneously associated with four ASE SNP markers of the *CMYA5* gene, and the rs110663707 aseQTL was associated with three SNPs, also of the *CMYA5* ASE gene. Moreover, 54 aseQTLs were associated simultaneously with different SNP pairs.

**Figure 2:**
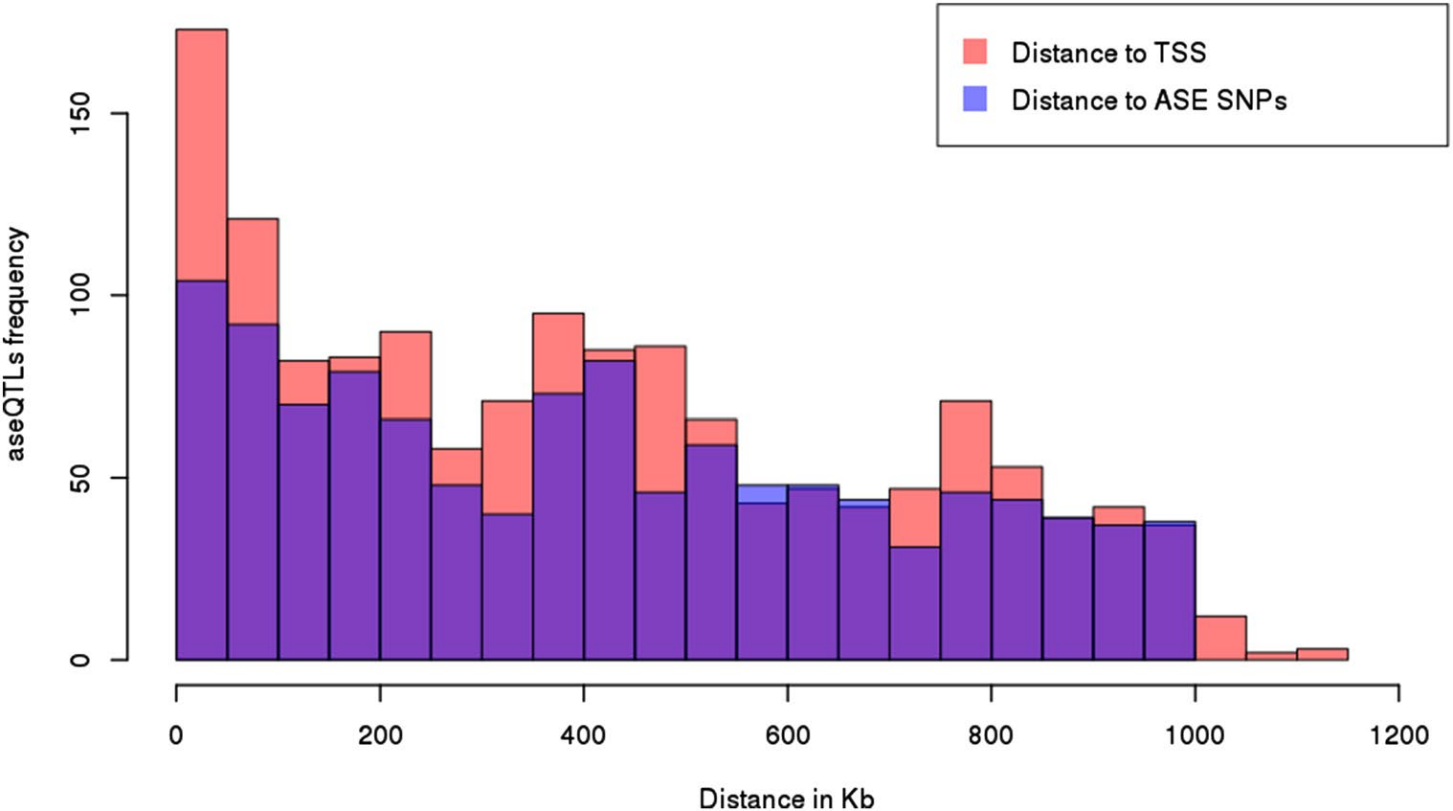
Distribution of aseQTLs concerning the distance to each associated ASE gene’s transcription start site (TSS) in kb (in pink) and each associated SNP in kb (in blue). X-axis: distance in kb. Y-axis: frequency of aseQTL. Histogram plotted using R basic functions.

An average distance of 404,983 bp (varying from 281 bp to 1.1 Mb) was observed between aseQTLs and the respective transcription start sites (TSSs) of affected ASE genes. Moreover, the average distance between an aseQTL and its associated SNP marker for ASE was 417,698 bp (ranging from 2238 bp to 999,650 bp). AseQTLs were more frequently located close to the TSS of ASE genes and their associated SNPs (from 0 to 100 kb), becoming less frequent as the distance increases, especially in intervals larger than 1 Mb. Figure 2 shows aseQTLs distribution compared with the ASE SNPs and the TSSs of genes showing ASE in their transcripts.

### Linkage disequilibrium analysis

**Figure 3:**
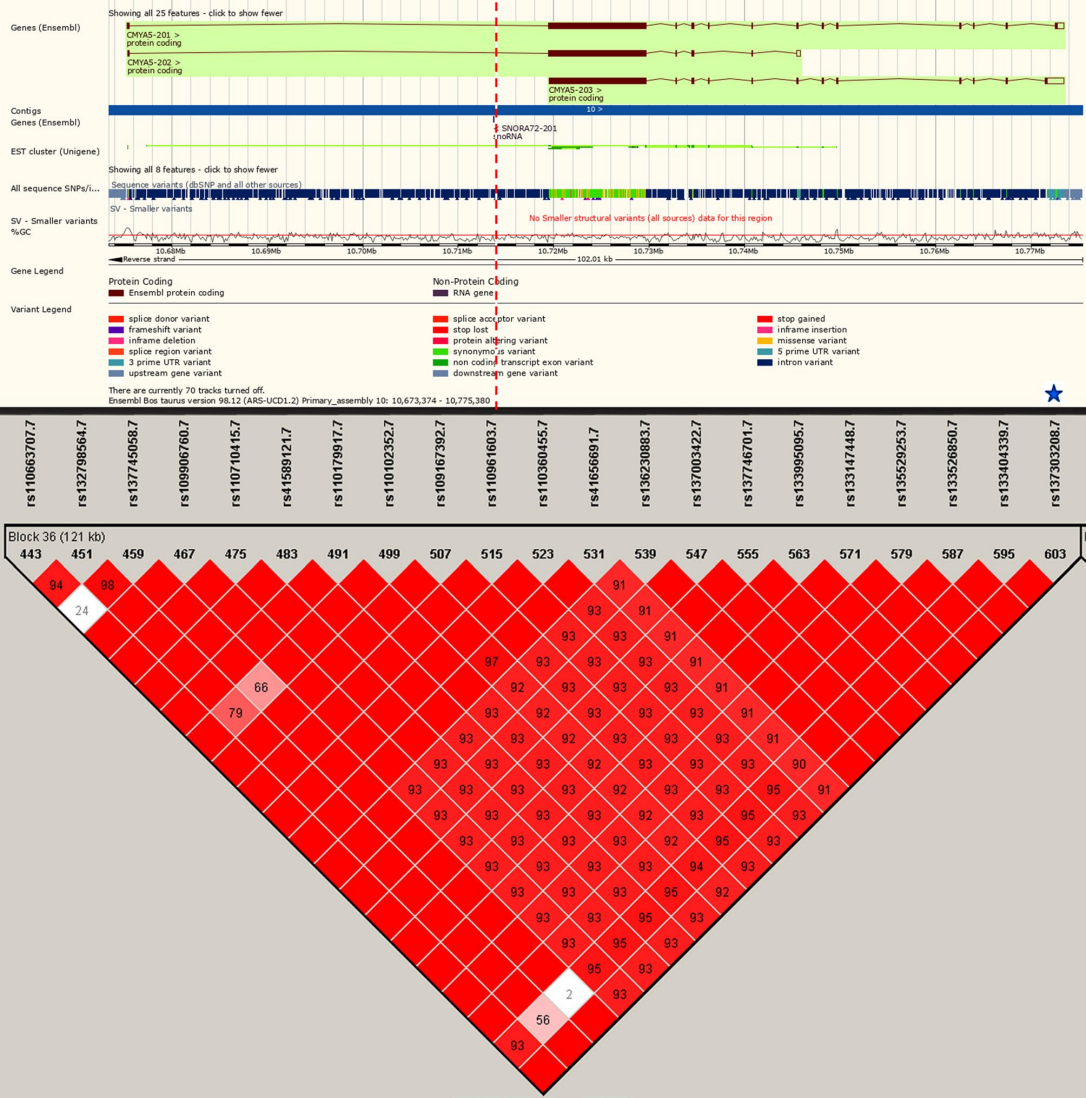
The biggest aseQTLs-LD block, located on chromosome 10, containing 21 aseQTLs. Red squares without numbers are SNPs in total LD, and the numbers within them indicate the intensity of the LD from 0 to 99. The intensity of the reddish tones increases proportionally to the LD values. The genomic position of the SNP rs137303208 is indicated with the dashed red line. All the SNPs in the block were within miRNAs binding sites. The blue star indicates that the SNP rs137303208 also was identified within TFBS. Gene representation was obtained in the Ensembl database and the LD plot was made using Haploview^76^. The two graphics were joined without any scale.

Firstly, we evaluated the linkage disequilibrium (LD) between all aseQTLs to investigate LD blocks with associated regulatory elements. A total of 3030 aseQTL pairs showed D′ greater than 0.8, and 1813 of them were in total disequilibrium (D′ = 1). In our aseQTL data, we identified 117 LD blocks (Supplementary Table 2) that included between two to 21 aseQTLs. The largest LD block contained 21 aseQTLs (Fig. 3) and affected 5 SNPs marking the ASE of the *CMYA5* gene.

Then, we performed the same analysis considering all SNPs located in transcripts showing ASE to observe if LD should be regarded on accounting for redundant aseQTL affecting the same allelic transcript. A total of 32 ASE SNPs pairs showed D′ > 0.8, being 31 with D′ = 1, distributed in 11 LD blocks (Supplementary Table 3). Seven blocks consisted of two neighboring SNPs marking ASE within the same gene, i.e.,* ASB5*, *ATP1A2*, *TXNIP*, *NXN*, *MAP4*, *EIF5,* and *CAVIN4* genes. For the gene *XIRP2*, two ASE SNP pairs were within two LD blocks. Three SNPs inside unknown ASE genes were within the same LD block, whereas the larger LD block involved five SNPs, all of them inside the *CMYA5* gene.

Lastly, we evaluated LD values between SNPs marking ASE and their respective associated aseQTLs. From 1134 SNP pairs submitted to LD analysis, 238 showed D’ higher than 0.8, and 71 aseQTL/ASE SNP pairs were in total disequilibrium (D’ = 1) (Supplementary Table 4) corresponding to 35 ASE genes. The gene *CMYA5* was identified as presenting more aseQTL/ASE SNP interactions (38), followed by *EIF5* (33) and *VCP* (27).

### aseQTLs overlapping with QTLs

After converting the QTLs retrieved from the Cattle QTL Database to the current genome assembly build, ARS-UCD1.2, we identified overlaps of 173 aseQTLs within QTL regions for 21 traits, distributed in 215 occurrences (Supplementary Table 5). The permutation approach applied to test if the probability of the aseQTLs location within QTL regions was higher than chance, was significant (*P* value = 0.0001). The three traits with the highest number of aseQTLs within a QTL region were body weight (81 aseQTLs), milk palmitoleic acid content (42 aseQTLs), and intramuscular fat (IMF) (28 aseQTLs). The rs136717535 aseQTL overlapped with regions of more QTL traits: milk kappa-casein percentage, milk protein percentage, shear force, calf size, and birth index.

We also integrated data with other studies produced by our research group, with a broader sample of the same experimental population. All permutation tests performed for QTL ranges data were significant (*P* value ≤ 0.05). We identified 234 aseQTLs distributed in 894 overlaps with 25 traits (Supplementary Table 6). The aseQTLs that integrate with GWAS data from our research group are distributed in the blue ring of Fig. 1. Of these, we identified 847 overlaps with meat quality traits^21^. Table 1 shows the number of overlapping aseQTLs with QTLs associated with meat quality traits and the complete name of each trait.

**Table 1:** Allele-specific expression quantitative trait loci (aseQTLs) overlapping meat quality traits identified in Nelore muscle and allele specific expression (ASE) genes affected by aseQTLs.

Meat quality trait^a^	Number of aseQTLs	Affected ASE genes
Ribeye area (REA)	192	*ACOT13, ASB5, CA2, CAB39, CMYA5, CUEDC1, DNAJC21, ITGB1, KLF10, MBNL2, NXN, PTP4A3, RGCC, SPARC, STBD1, XIRP1, XIRP2*
Lightness of fat (L*FAT)	127	*ACOT13, ASB5, CAB39, CMYA5, DNAJC21, ITGB1, KLF10, NXN, SPARC, STBD1*
Warner–Bratzler shear force 7 days after slaughter (WBSF7)	122	*ACOT13, ASB5, CAB39, CMYA5, DNAJC21, ITGB1, KLF10, SPARC, STBD1*
Water holding capacity (WHC)	113	*ACOT13, ASB5, CAB39, CMYA5, DNAJC21, ITGB1, KLF10, SPARC, STBD1*
Cooking loss (CL)	105	*ACOT13, ASB5, CMYA5, DNAJC21, ITGB1, SPARC, STBD1*
Yellowness of fat (b*FAT)	100	*ACOT13, CAB39, CMYA5, DNAJC21, ITGB1, KLF10, SPARC, STBD1*
Warner–Bratzler shear force 24 h after slaughter (WBSF0)	46	*CMYA5, PFKM*
Lightness muscle (L*MUSCLE)	23	*ACOT13, SPARC,*
Backfat thickness (BFT)	17	*ACOT13, STBD1*
Warner–Bratzler shear force 14 days after slaughter (WBSF14)	2	*SPARC*

The rs109550233 SNP, marker of allelic imbalance, presented more associated aseQTLs overlapping with QTLs for meat quality traits than any other in this study, with nine aseQTLs within regions of QTLs for nine traits, b*FAT, BFT, CL, L*FAT, L*MUSCLE, REA, WBSF0, WBSF7, and WHC, followed by the rs135906938 SNP (*ACOT13* gene), with eight aseQTLs in QTLs for eight meat quality traits, i.e., b*FAT, BFT, CL, L*FAT, L*MUSCLE, REA, WBSF7, and WHC*.* Thirty-five aseQTLs associated with six SNPs marking ASE of *CMYA5* overlapped with seven meat quality traits: b*FAT, CL, L*FAT, REA, WBSF0, WBSF7, and WHC.

Ten aseQTLs overlapped with mineral content QTLs for Co, Mn, Zn, Ca, Cr, Ar, K, Mg, and S^24^; seven overlapped with QTLs for IMF composition, which were also associated with octadecenoic acid^25^; Six overlapped with feed efficiency associated traits: efficiency of gain (EG), maintenance efficiency (ME) and partial efficiency of growth (PEG)^26^. Additionally, 20 aseQTLs overlapped with *cis*-eQTL regions and four with trans-eQTL regions^27^.

### Annotation of the aseQTL SNPs

According to aseQTL locations in the genome, we predicted SNP consequences with the VEP software^28^ (Supplementary Table 7). AseQTLs were predominantly distributed in intronic regions (68%), followed by intergenic regions (27%). The remaining approximately 5% were distributed equally in non-coding transcription regions, 3′UTR variants, missense variants, and synonymous variants. Four SNPs, rs132671408 (in the *AOPEP* gene), rs135305605 (in the *RUSC2* gene), rs136155631 (in the *RAI14* gene) and rs43291678 (in the *CCDC141* gene) were within the 5′UTR regions and three were located in splice regions: rs109091165 (inside *PECAM1* gene), rs133325919 (inside *FAIM2* gene) and rs137526254 (inside *MYO6* gene).

### Methylated aseQTLs

**Figure 4:**
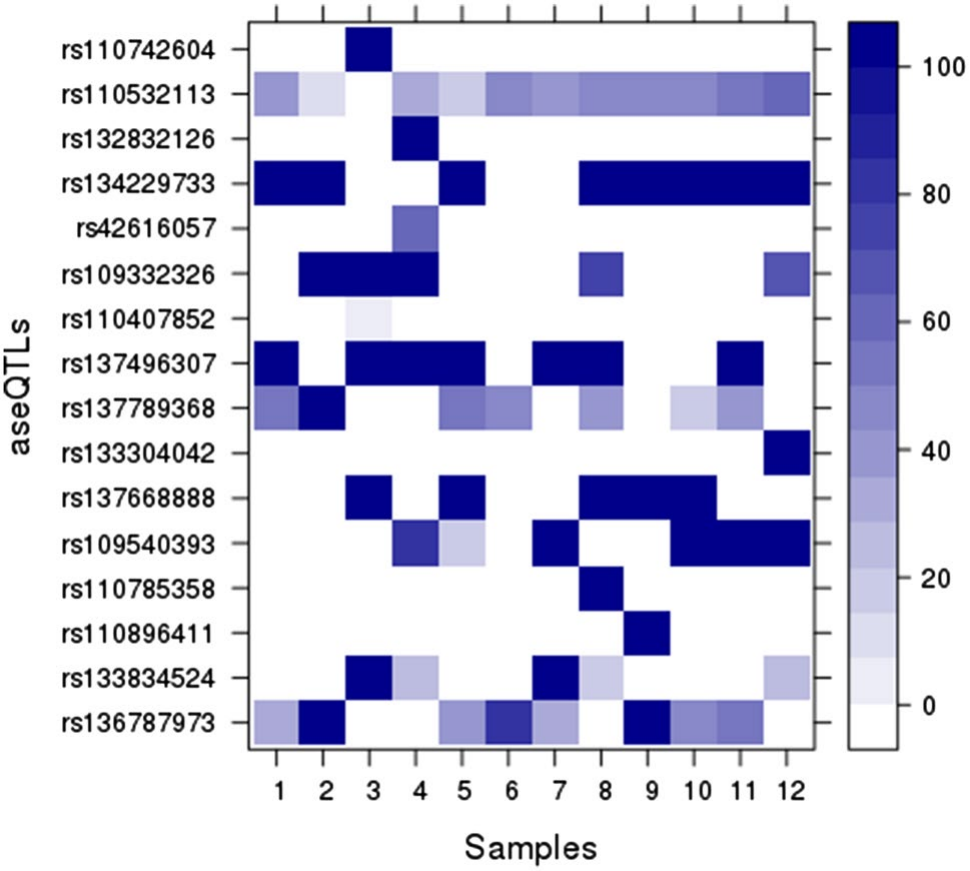
Percentage of aseQTLs methylation in 12 Nelore animals. The color intensity increases proportionally according to the methylation percentage for each SNP per sample. Heatmap plotted with Lattice R package (http://lattice.r-forge.r-project.org/).

To analyze the methylation pattern, we compared the positions of aseQTLs with the methylation percentage of SNPs identified by RRBS in 12 samples. We identified 69 methylated cytosines in 16 aseQTLs, whose distribution is shown in the purple ring of Fig. 1. The rs110532113 aseQTL, associated with an ASE SNP on the *MSRB1* gene, was methylated in 11 of the 12 tested animals. Thirteen aseQTLs had 37 cytosines with 100% of methylation in the samples. Eight animals showed 100% of methylation in the rs134229733 aseQTL, and seven animals in the rs137496307 aseQTL, associated with an SNP marker of ASE of the *VIM* gene (Fig. 4; Supplementary Table 8).

Six methylated aseQTLs were associated with SNPs inside transcribed regions with the allelic imbalance of seven genes: *HSPA1A*, *MSRB1*, *VCP*, *VIM*, *SCN4A*, *PECAM1,* and *ZDHHC4*, being the rs137668888 aseQTL simultaneously associated with the ASE marked by SNPs of *SCN4A* and *PECAM1* genes. The rs110407852 was the methylated aseQTL closest to the TSS region of the respective associated gene (*VCP*), at a distance of 51,026 bp from it. The average distance of these methylated aseQTLs and the TSS of associated ASE genes was 216,169 bp. The other ten methylated aseQTLs were associated with 9 ASE SNPs. The rs42616057 and rs109332326 aseQTLs were both methylated and associated with the allelic imbalance of rs110095343 SNP.

### aseQTLs within TFBS

Using 50 bp flanking sequences of all aseQTLs as input in TRAP software^29^, we found 83 TFBS overrepresented in our sequences (FDR < 0.05, Supplementary Table 9). Of them, only 12 TFs are expressed in bovine muscle^30^. Considering only aseQTLs that change muscle-expressed TFBS, we found 215 aseQTLs, distributed in the pink ring of Fig. 1. From these, 150 presented putative TFBS with the reference allele and 117 with the alternative allele. The SMAD4 binding site was found affected by 37 aseQTLs; AHR by 31 aseQTLs; VDR and SP3 by 26 aseQTLs; STAT3 by 25 aseQTLs; PPARA by 24 aseQTLs; DR1, TAL1, and ZNF219 by 20 aseQTLs; STAT6, SP1, and MZF1 TFs were affected by 16, 12, and 10 aseQTLs, respectively.

### aseQTLs within miRNA binding sites

We used a subset of 163 aseQTLs identified outside intergenic regions and that overlapped with our reference population’s QTL data for miRNA binding site prediction. We identified 153 miRNA binding sites corresponding to 163 aseQTLs flanking sequences. Considering miRNA binding sites affected by aseQTL, we obtained 1448 aseQTLs/miRNA binding site pairs. In silico analysis of these pairs predicted 693 miRNA binding sites with the alternative aseQTL allele, whereas 755 binding sites were predicted in the reference aseQTL allele (green ring of Fig. 1). The pairs were formed with 162 aseQTLs and 149 miRNAs, being 114 miRNAs expressed in the muscle of our population^31^. These muscle-expressed miRNAs were denoted without an asterisk in Supplementary Table 10, which contains the results of miRNA binding sites prediction.

We analyzed whether the ASE genes were previously identified as targets for the miRNAs affected by the associated aseQTLs with the MiRWalk tool^32^. Twenty genes associated with aseQTLs that potentially change miRNA binding sites' affinity were used in the MiRWalk analysis. These genes were targets for 583 miRNAs. From these, 31 miRNAs had their binding sites affected by the aseQTLs predicted to regulate 14 ASE genes, which were themselves identified as targets for the corresponding miRNAs.

In the following examples, aseQTLs affected the binding sites of miRNAs known to affect the ASE gene in question. The rs42437277 aseQTL, associated with the allelic imbalance of the *ASB5* ASE gene, was predicted to affect the binding of five miRNAs. The rs133661649 aseQTL, associated with an SNP showing ASE of the *LPL* gene, and the rs109408013 aseQTL, associated with the ASE SNP of *NXN* gene, were both targets of three miRNAs. Seven aseQTLs were affecting two miRNA binding sites that potentially regulate the associated genes: rs41255633 (*NXN*), rs135834852 (*NXN*), rs136463427 (*CUEDC1*), rs110417686 (*CUEDC1*), rs132705216 (*MBNL2*), and rs137489440 (*MBNL2*), rs133710221 (*CAB39*). The complete list of aseQTLs affecting the binding sites of miRNAs known to target the ASE genes is in Supplementary Table 11.

### Regulatory mechanisms summary

**Figure 5:**
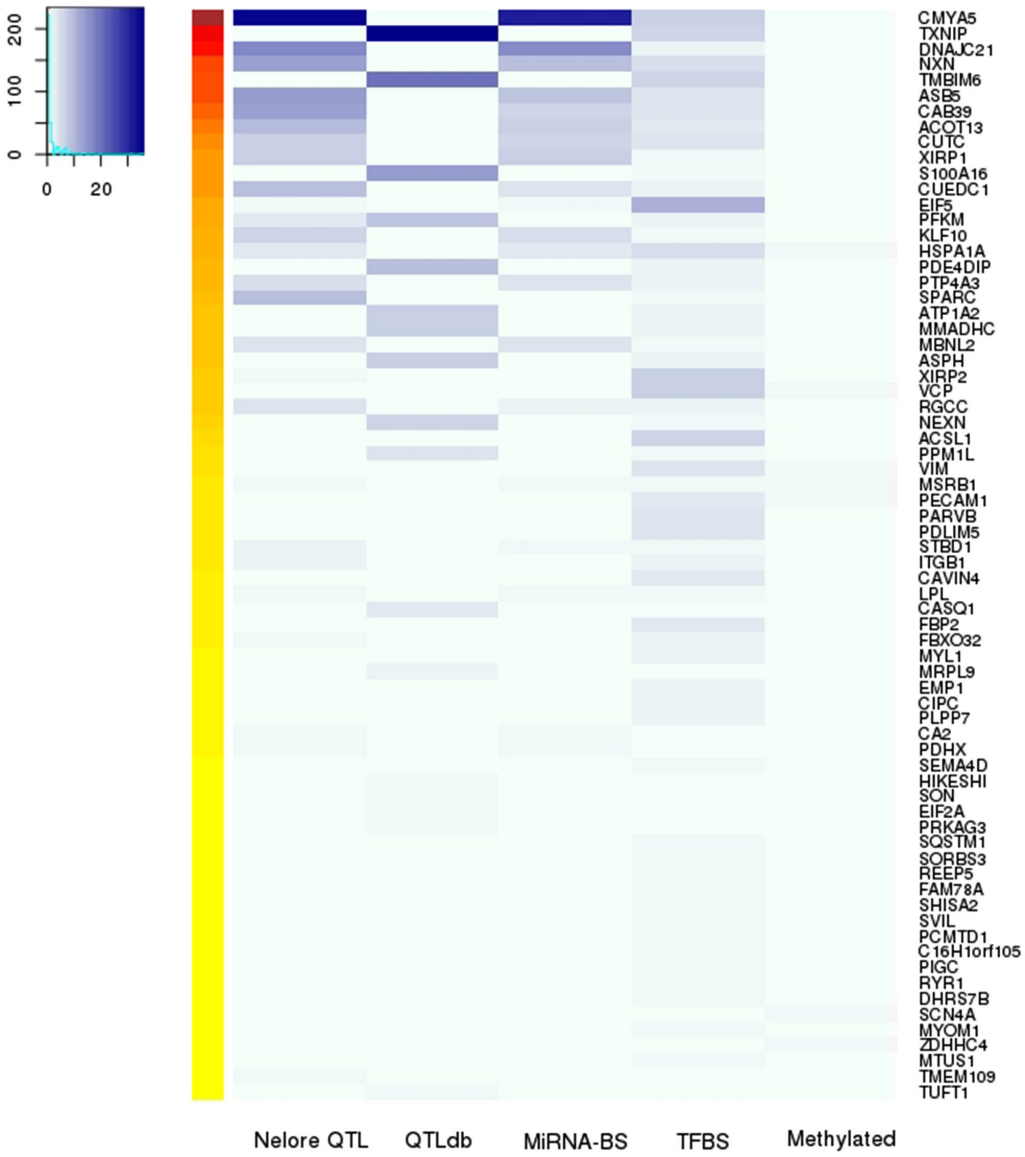
Heatmap of the aseQTLs distribution according to the associated gene and the regulatory regions predicted here. In the rows are the ASE genes for which we found at least one aseQTL inside a regulatory region. Columns represent “results sections”: Nelore QTLs have the number of aseQTLs that overlapped with Nelore GWAS data; QTLdb has the aseQTLs in QTLs regions from Cattle QTL Database; miRNA-BS, TFBS, and methylated columns have the aseQTLs that potentially changes miRNA binding sites, transcription factors binding sites and that was methylated. The blue scale increases according to the presence of aseQTLs in each results section. The left heatmap with hot colors represents the total of regulatory regions that combine with our aseQTL data for each gene. The figure was created with Gplots R package (https://github.com/talgalili/gplots).

In short, we predicted three regulatory mechanisms in our aseQTL data: methylation, TFBSs, and miRNA binding sites. Only one aseQTL, rs109540393, associated with the ASE present in the rs109550233 SNP genomic location, was methylated in six animals of our population and was potentially creating the binding site of the AHR TF. Six miRNAs expressed in Nelore muscle had binding sites created by the aseQTL rs109540393, five matching the reference allele sequence and one the alternative allele. This aseQTL overlapped with nine meat quality traits. Moreover, 33 aseQTLs were within two regulatory mechanisms, 354 were predicted to have one regulatory mechanism, and 746 did not show any regulatory evidence. Supplementary Table 12 contains the number of regulatory mechanisms or overlapping QTLs for each aseQTL. In Fig. 5, we integrate all our results to identify interesting ASE genes with associated aseQTLs in regulatory regions. The top 10 ASE genes, according to the number of aseQTLs with relevant attributes, ranging from 73 to 15 aseQTLs, were *CMYA5, TXNIP, DNAJC21, NXN, TMBIM6, ASB5, CAB39, ACOT13, CUTC,* and *CUEDC1*, respectively. Considering each section of results, represented in Fig. 5 as columns, the top gene for each category was: *CMYA5*, with 35 aseQTLs overlapping seven meat quality traits identified in our Nelore population; *TXNIP*, with 36 aseQTLs located in QTL regions from Cattle QTL Database; *CMYA5* with 31 aseQTLs potentially changing the binding of miRNAs and *EIF5* gene showed 11 aseQTLs in TFBSs. Additionally, seven genes showed one methylated aseQTL.

## Discussion

In this study, we identified 1134 aseQTLs associated with the ASE pattern of 126 SNPs in the *Longissimus thoracis* muscle of Nelore steers. Due to the sample requirement to associate each SNP marking ASE and aseQTL SNP, we tested 23% (192 out of 820) SNPs with significant ASE previously detected in the same population^5^ and identified aseQTLs for 126 (66%) of them. Thirty-seven ASE SNPs associated with aseQTLs were also described in a previous ASE study developed with bovine muscle samples^8^. Two ASE SNPs described by Guillocheau et al. (2019) were identified here as aseQTLs: rs109170993, associated with the rs110850310 ASE SNP, and rs109842586, associated with the rs109372848 ASE SNP. The rs109842586 was also identified marking ASE in our population as well^5^.

Sixteen percent of our aseQTL data overlapped with QTL regions from the Cattle QTL Database, showing enrichment for milk quality and production traits, although we must consider a bias in the frequency of publications for each trait in that database. The rs136717535 aseQTL, which presents more overlapping QTLs from Cattle QTL Database, including for shear force, was associated with the ASE SNP rs41257152. This SNP is located in the *HIKESHI* gene, which mediates heat stress-induced nuclear transport of Hsp70 heat shock proteins, protecting cells from heat shock damage^33,34^. Nelore cattle are widely valued by their heat tolerance^35,36^, and this aseQTL potentially regulates a heat stress-related gene’s allelic expression. Moreover, the Hsp70 protein had a lower abundance in more tender muscle^37^ in the Nelore population used in this study, thus reinforcing this aseQTL as a potential regulatory variant for shear force in Nelore. On the other hand, comparisons with our Nelore population QTL data revealed enrichment for meat quality traits, with 94.7% of the overlapped aseQTLs located in regions of 10 meat quality phenotypes^21^. For example, *SPARC*-associated aseQTLs overlapped with lightness and yellowness of fat, WBSF7, and WBSF14, being this gene identified as differentially expressed between contrasting samples for IMF content^27^ in our population, and also classified as a potential target for IMF and tenderness in swine^38,39^. Seven QTL regions, including for BFT, b*FAT, and L*FAT, overlapped with aseQTLs associated with the allelic imbalance of an SNP of the *STBD1* gene, which was differentially expressed between contrasting samples for IMF content^27^. Additionally, *CMYA5* had 245 overlaps between aseQTLs and QTLs for b*FAT, CL, L*FAT, REA, WBSF0, WBSF7, and WHC traits. In addition, ASE genes in the bovine muscle functionally enriched for meat quality traits were previously identified in our population^5^ and elsewhere^8^. Although these overlaps with QTLs might be useful for prioritizing SNPs for validation, a word of caution is necessary, as the confidence intervals for the QTLs might be considerable and could not be taken into account in our analyzes.

As expected, most aseQTL associations with more than one SNP marking ASE were with ASE SNPs blocks that presented total LD (D′ = 1), suggesting that these aseQTLs haplotypes regulate the same transcript. For example, 14 aseQTLs located in two LD blocks were associated with 2 SNPs, displaying total LD. These SNPs marked the allelic imbalance of the *EIF5* gene, previously associated with marbling in Nelore cattle^40^. The rs109922571 aseQTL had strong LD among other SNPs in these two LD blocks and was predicted to affect miRNA and TF binding sites. Another example was the rs132798564 aseQTL, associated with four SNPs in the transcribed ASE regions of the *CMYA5* gene, with total disequilibrium (D′ = 1) within their block. Additionally, 15 aseQTLs were associated with the ASE of two different pairs of *CMYA5* SNPs, each of them in complete LD (D′ = 1). *CMYA5* was associated with IMF in pigs^41^. It lies within regions associated with REA in Nelore cattle^40^ and six other meat quality traits^21^. This gene encodes for a desmin-associated protein^42^, which can regulate vesicular/lysosome trafficking^43^. The autophagy-lysosome pathway is a degradation system involved in muscle wasting and muscular atrophy^44^. Moreover, CMYA5 protein may protect from autolysis^45^ the CAPN3, whose variants were associated with meat tenderness in zebu cattle^46^. The aseQTLs distributed in LD blocks within the *CMYA5* gene may be affecting the allelic expression of this gene. The most informative SNP, displaying strong LD with all SNPs of the largest LD block, is located in a specific intronic region present in two out of three *CMYA5* isoforms. Thus, its enrolment on *CMYA5* splicing regulation should be further investigated. Our results indicate that LD analysis is essential for the interpretation of ASE association tests in the search for causal mutations because most of the SNPs marking ASE and their associated aseQTLs could be reduced to LD blocks, which in turn makes it difficult to pinpoint the causative mutation.

Both the LD results and the presence of aseQTLs nearby the respective TSS or close to the associated ASE SNP were indicatives of potential *cis*-regulatory activity of aseQTLs. *Cis*-eQTLs and aseQTLs were identified more frequently near the TSS of affected genes^17,47,48^. AseQTL annotation showed similar results as *cis*-regulatory SNPs obtained in the same population^27^, from which twenty *cis*-eQTLs overlapped with our aseQTL data. The predominant distribution of aseQTLs and *cis*-eQTLs in intergenic and intronic regions supports the concept that the SNPs in these genomic locations tend to have a regulatory role^49,50^.

*Cis*-acting variants can modify TFBSs^51^, resulting in the allele-specific loss of TF binding^52^ and leading to ASE in heterozygous animals. We found 215 aseQTLs within 12 TFBS for TFs expressed in bovine muscle^30^. Binding sites for five different TFs were predicted to be affected by more than one aseQTL: SMAD4, AHR, VDR, STAT3, and PPARA. All these TFs have been associated with phenotypes related to muscle tissue. SMAD4 deficient knockout mice showed muscle atrophy^53^, PPARA was related with lipid metabolism^54^, VDR is a vitamin D receptor that was suggested to regulate marbling and calcium homeostasis^55^, the AHR TF was associated with human aging^56^, STAT3 is related to muscle wasting^57^ and muscle regeneration^58,59^. The alternative allele of the rs135348406 aseQTL was identified within a STAT3-binding site and was associated with an SNP marking ASE of *HSPA1A* gene, which encodes the Heat shock 70 kDa protein 1. STATs are known to modulate heat shock proteins, including Hsp70^60^. The reference allele of the rs137692633 aseQTL was predicted to benefit the binding of STAT3 and STAT6 transcription factors. Besides that, SP1, SP3, VDR, and ZNF219 TFs were predicted to have a binding affinity with the alternative allele of the same aseQTL.

Epigenetic mechanisms, as DNA methylation^2,11^, can also be associated with ASE^8,61^, repressing the transcription of a specific hypermethylated allele^11^. To identify whether the methylation of these aseQTLs could have an important role in ASE, we compared the level of allelic imbalance ratio of the ASE SNPs with the methylation of the associated aseQTLs per animal. However, in our study, only a few animals with methylation data showed significant ASE in an SNP associated with a methylated aseQTL. The methylated aseQTL closest to the TSS of the respective SNP marking ASE (within *VCP* gene) was rs110407852, with methylation in the alternative allele. The corresponding ASE SNP was monoallelic in nine animals, expressing only the allele in phase with the reference aseQTL allele, thus providing an indication of methylation-mediated silencing of the allele phased with the methylated aseQTL. Similarly, three animals with the genotype 1|0 for the ASE SNP rs42719199 showed an allelic imbalance ratio of 0.5, expressing only the alternative allele. The associated rs136787973 aseQTL showed methylation rates of 32.30%, 80%, and 55.75% and the genotypes C|T, T|C, and T|C, respectively. Thus, it could be postulated that, for the two animals with higher methylation levels for this aseQTL (80% and 55.75%), the T|C aseQTL genotypes and the methylated cytosine potentially silenced the expression of the ASE SNP reference allele, which should be further investigated. We considered that analyzing the methylation pattern in individual candidate variants was of little value on indicating the causative variants, probably due to our restricted methylation data.

As well as methylation, miRNAs^62,63^ have been related to genomic imprinting. Some studies related the allelic imbalance with the *cis-*regulatory action of miRNA binding sites^64,65^, which was also described in bovine muscle^8^. We identified 162 aseQTLs within miRNA binding sites, potentially changing the binding affinity of 149 miRNAs, distributed in 1448 miRNA/target pairs. Among the affected, the bta-miR-423-5p and bta-miR-486 miRNAs were differentially expressed between extreme groups for IMF^31^ and RFI^66^, respectively, in our reference Nelore population. Fourteen ASE genes affected by aseQTLs within binding sites of miRNAs were predicted as targets for the corresponding miRNA. For instance, the rs42437277 aseQTL, associated with the ASE marked by an SNP in the *ASB5* gene, was predicted to affect the binding of five miRNAs which, in turn, are predicted to target this gene. Thus, the integration of our data and the known miRNA-target genes information offers more reliability for these findings.

Some aseQTLs appeared more than others among the analyzes performed in this work. The rs109540393 aseQTL, which overlapped with nine meat quality QTLs identified in our population^21^, was methylated, predicted to affect a binding site of six muscle-expressed miRNAs and the TFBS of AHR. AHR was negatively associated with aging^56^ and angiogenesis^67^, although the associated allelic imbalance SNP marker is within an unannotated transcript. Finally, the *CMYA5* associated aseQTLs, which showed more overlaps with Nelore QTLs (35), contained a larger number of aseQTLs affecting miRNA binding sites (7), including sites for two differentially expressed miRNAs for IMF and RFI. The combined results demonstrate that muscle-related aseQTLs are located in regions associated with bovine traits, mainly enriched for meat quality QTLs, including REA, tenderness, and IMF. Tenderness is an essential factor for consumers’ acceptance^6,7^, while IMF is related to palatability and juiciness^68^, sensorial characteristics that affect consumer choice and meat price^69^. Some genes seem to have a strong aseQTL regulation, influenced by different and integrative *cis*-regulatory mechanisms, as *CMYA5*, which the literature indicates as functionally relevant for meat quality phenotypes. These results suggested that the aseQTLs regulating the *CMYA5* gene can be candidates for the improvement of sensory traits of Nelore beef.

Thus, our results reinforce a complex regulation of ASE in bovine muscle, indicating potential *cis-*regulatory variants whose effects remain to be explored due to the high LD and the lack of conclusive evidence about causal variants. As we were not able to address false positives due to the restrictions of sampling, these results should be interpreted along with other evidence for regulatory function for each given potential aseQTL. Future studies may prioritize SNPs based on aseQTLs for experimental validation, and methodological approaches can be designed according to the potential regulatory mechanisms and phenotypes predicted in this work.

## Material and methods

### Use of experimental animals

The study complied with the ARRIVE guidelines. All experimental procedures and animal protocol were carried out following the relevant guidelines and regulations provided by the Institutional Animal Care and Use Committee Guidelines of Embrapa Pecuária Sudeste ethics committee (São Carlos, São Paulo, Brazil. Protocol CEUA 01/2013). The Ethical Committee of Embrapa Pecuária Sudeste (São Carlos, São Paulo, Brazil) approved all experiments (approval code CEUA 01/2013).

### Sample collection

Details regarding the animal's production can be found in Tizioto et al.^21^ Concisely, we used a subset of the described experimental population, consisting of 190 Nelore steers. These animals were sired by artificial insemination using 34 bulls that represented the main lineages of the breed, monetarily accessible to any Brazilian producer and not closely related. The steers were raised until 18 months of age in three farms and afterward maintained in three feedlots under identical nutrition at Embrapa Pecuária Sudeste (São Carlos, São Paulo, Brazil). Animals were slaughtered according to the subcutaneous fat thickness (5 mm), with an average weight of 383.2 kg and around 24 months of age.

### DNA extraction and genotyping

The DNA was extracted from blood samples by using salting out method^70^. DNA concentration and purity were measured by spectrophotometry in NanoDrop. Integrity inspection was made by agarose gel electrophoresis.

Genotyping was performed with Illumina BovineHD BeadChip (Illumina Inc, San Diego, CA, USA) at the USDA ARS Bovine Functional Genomics Laboratory in Beltsville, MD, USA and the ESALQ Genomic Center in Piracicaba, SP, Brazil^71^. Quality control was performed with PLINK software^72^ from the autosomal SNPs with genome coordinates corresponding to the bovine reference genome (Assembly: ARS-UCD1.2). SNPs with MAF lower than 5%, as well as samples and SNPs with a call rate lower than 95%, were discarded. In addition, we removed SNPs with a Hardy–Weinberg Equilibrium *P* value ≤ 0.0001. This genotype file has a total of 429,513 SNPs available for ASE analysis. The genotypes were phased using BEAGLE software^73^ with default configurations. We did not use imputation parameters and did not use a pedigree file, so all the samples were considered unrelated individuals.

### RNA extraction and sequencing

A total of 100 mg of *Longissimus thoracis* tissue from 190 animals were collected immediately after slaughter, submerged in liquid nitrogen, and stored at − 80 °C until RNA extraction. Total RNA extraction and RNA sequencing methodology were described elsewhere^27^. Briefly, RNA extraction was made using TRIzol reagent (Life Technologies, Carlsbad, CA, USA), following the manufacturer’s instructions. Bioanalyzer 2100 (Agilent, Santa Clara, CA, USA) was used to verify the RNA integrity and the TruSeq RNA Sample Preparation kit v2 guide (Illumina, San Diego, CA) was used to prepare the RNA libraries from 2 µg of RNA. We also used the Bioanalyzer 2100 to determine the average size of the libraries and the quantitative PCR with the KAPA Library Quantification kit (KAPA Biosystems, Foster City, CA, USA) to carry out the quantification. Clustering and sequencing were performed with Illumina HiSeq 2500 (Illumina, San Diego, CA, USA), and SeqyClean software (https://github.com/ibest/seqyclean) was used to remove sequencing adapters and low complexity reads. The quality control was performed with FASTQC *software* version 0.10.1 (https://www.bioinformatics.babraham.ac.uk/projects/fastqc/). The sequencing was performed at the Genomics Center at ESALQ, Piracicaba, São Paulo, Brazil, and archived on the European Nucleotide Archive (ENA) under accessions: PRJEB13188, PRJEB10898, and PRJEB19421.

### ASE analysis

SNPs located in regions showing ASE were obtained from De Souza et al. (2020). In brief, the authors identified heterozygote SNPs from Illumina BovineHD BeadChip genotyping data, and the genotypes were phased and organized in individual files. Together with the bovine reference genome (ARS_UCD1.2), these files were used as inputs in the ALEA algorithm^74^, which created two diploid homozygous genomes basing on the two haplotypes from each heterozygous animal. After that, the RNA-Seq reads of the 190 steers were aligned to each in silico-created genome, resulting in the counts of reads that mapped the first or second haplotype per SNP and individual. A binomial test was applied to determine if allele counts were statistically divergent for each SNP and animal. Finally, a total of 820 SNPs in ASE (FDR < 0.05) were described and used in this study^5^.

### aseQTLs identification

For the ASE-association test, we selected SNPs within 1 Mb upstream and downstream from each SNP marking ASE identified previously^5^ because this window probably encompasses *cis*-regulatory SNPs. To test for the presence of aseQTLs, the mean values of allelic expression imbalance, i.e., the ratio of counts from each allele, were computed and compared between heterozygote and homozygote animal groups for the candidate aseQTL, using the non-parametric Wilcoxon Rank Sum test (P-value ≤ 0.05)^17^. If an SNP has regulatory action on the allelic expression, we only can observe ASE when the genotype is heterozygous. If this SNP is in homozygosis, the two alleles will contribute equally to allelic expression. Thus, the identification of an aseQTL occurs if animals of the heterozygous aseQTL group had an imbalance of allelic expression (present in the SNP marking ASE) significantly higher than any homozygous aseQTL group, being always tested with the homozygous group with more animals. This analysis required a minimum of 10 heterozygous animals for each test, both in candidate’s aseQTLs and ASE SNPs.

The aseQTLs identification methodology comprises a non-parametric test and requires a large number of heterozygotes, which makes it more restrictive, being more rigorous for an association to be significant. The required minimum repetition of genotypes in the population increases confidence for the results, but unfortunately, such conditions turn unviable the correction for multiple tests due to a large number of tests for few significant results. As the aseQTLs are candidate SNPs for the understanding of bovine regulation, thus to be further verified by other methods, we followed the method described previously^17^ and, as in that reference, we did not apply any method for multiple comparisons correction.

The aseQTL allele frequency was computed with VCFtools software^75^. To compare aseQTL positions with the TSS, we extracted from BioMart (Ensembl Release 98. Access in: https://www.ensembl.org/biomart/martview) the TSS positions for all annotated ASE genes. AseQTLs SNPs were annotated using the Variant Effect Predictor (VEP, Ensembl Release 98) software^28^.

### Linkage disequilibrium analysis

Some associations between SNPs may result from population dynamics, such as the presence of LD by calculating D’ statistics. We choose D’ statistics because it is less sensitive to allele frequency than R^2^ and we aimed to inspect any possible LD, including rare variants. We calculated LD to investigate allele relationship among aseQTLs and SNPs located in ASE regions in three ways using the software Haploview^76^: (1) LD analysis between aseQTLs and their respective ASE SNPs (to verify if the high LD is relevant for ASE-association test significance); (2) LD analysis between all aseQTLs (to investigate possible ambiguous results and indicate conserved *cis*-regulatory elements within an LD block); and III) LD analysis among the SNPs marking ASE (to understand whether the same aseQTLs can be associated with more than one ASE SNP because of high LD).

### The overlap between aseQTLs and GWAS data

We intersected data from aseQTLs with QTL regions previously associated with economic traits for beef cattle. For an overview on how aseQTLs can affect phenotypes of interest in other populations, the overlap of each aseQTL position was intersected with QTL positions obtained from the Cattle QTL database (Assembly: UMD_3.1) (https://www.animalgenome.org/QTLdb/cattle/ downloaded in June 2019).

AseQTLs were integrated with data described by our research group for eQTL^27^ and QTLs, using a larger population that comprised the animals used here, identified by GWAS of meat quality traits^21^, mineral content^24^, feed efficiency^26^, and IMF content^25^. For the integration with position ranges of QTLs, a permutation test was performed with 1000 iterations using RegioneR package^77^ to investigate the randomness of aseQTL locations within these potential regulatory regions. In both QTL overlap analyses, we could not correct for a confidence interval of QTLs since this information was not available.

All the GWAS data obtained by our research group or in the Cattle QTL database in studies performed with the UMD 3.1.1 reference genome was converted for the ARS-UCD1.2 version using Lift Genome Annotations UCSC tool (https://genome.ucsc.edu/cgi-bin/hgLiftOver).

### Methylation profile

To determine the methylation profile in Nelore muscle, we subset 12 animals of our population for the Reduced Representation Bisulfite Sequencing (RRBS), selected based on the ranking of the estimated breeding value (EBV) for the tenderness phenotype^78^. The muscle samples were collected as described before, and DNA extraction was made by using DNeasy Blood & Tissue kit (Qiagen, Hilden, Germany), according to the manufacturer's protocol. DNA concentration was quantified with Qubit dsDNA High Sensitivity Assay (Thermo Fisher Scientific). The quality was measured with Fragment Analyzer and DNF-487 Standard Sensitivity or the DNF-488 High Sensitivity Genomic DNA Analysis *Kit* (Advanced Analytical).

The RRBS experiments were performed by Diagenode. Each sample library was prepared from 100 ng of genomic DNA using the Premium RRBS kit (Diagenode), according to the manufacturer's protocol. Bisulfite conversion efficiency independent of the CpG context was assessed by adding methylated and unmethylated spike-in controls (concentration of 0.1%). In summary, the protocol consisted of the digestion of DNA by the MspI enzyme, followed by the fragment end repair and addition of adaptors. Thus, samples were quantified by qPCR, being the Cq values used to pool samples by similarity. Bisulfite conversion was performed using the Premium RRBS kit (Diagenode) according to the manufacturer's protocol, followed by library enrichment by PCR. Adequate fragment size distributions were confirmed by Bioanalyzer High Sensitivity DNA chips (Agilent).

Libraries were sequenced on Illumina HiSeq 3000 using single-end 50 bp reads. Sequencing read quality control was performed using FastQC version 0.10.1 (https://www.bioinformatics.babraham.ac.uk/projects/fastqc/), and adaptors were removed by Trim Galore! Version 0.4.1. (http://www.bioinformatics.babraham.ac.uk/projects/trim_galore/). Bismark version 0.16.1^79^ was used to align the reads to the *Bos taurus* reference genome ARS-UCD1.2 and to identify methylated cytosines.

The methylation profile was calculated by the percentage of methylation in a given region, dividing the number of methylated cytosines in that region by the sum of the methylated and unmethylated cytosines, multiplied by 100.

### Regulatory mechanism prediction

We performed three different approaches to investigate whether the aseQTL had additional evidence for being considered as the causal SNP affecting the allele-specific expression and predict possible regulatory mechanisms involved. First, we checked the methylation percentage in aseQTLs by integrating the exact positions of the aseQTLs and the methylated SNPs, which data was resultant from the RRBS experiment. Then, we compare if the animals with methylated aseQTL showed significant ASE^5^ to observe the existence of an interaction between the methylation and the allelic imbalance in that animal.

The aseQTLs with TFs affinity were identified using TRAP software (Transcription Factor Affinity Prediction) for multiple sequences^29^. The analyses were performed using the flanking sequences considering a window of 25 bp downstream and upstream to the aseQTL, with data from Transfac 12.1 Metazoans matrix and background model of chordate conserved elements. The resulting TFs were overlapped with a list of manually curated TFs expressed in bovine muscle^30^. We considered significant interactions whether the corrected P-values were ≤ 0.05 (with the Benjamini–Hochberg correction). We selected aseQTLs that changed the TF binding with the reference or alternative allele.

The third approach was to find aseQTLs overlapping predicted miRNA binding sites. To filter all the aseQTLs dataset for the miRNA binding site prediction, we used the aseQTLs outside of intergenic regions that integrate with other QTL regions identified in our population. The prediction of miRNA binding sites in our aseQTLs was made with the RNAhybrid software^80^. RNAhybrid calculates the most energetically favorable hybridization between the mRNA and miRNAs sequences. Here we evaluate the interaction between aseQTL flanking sequences and bovine mature miRNAs sequences available in the MiRBase database^81^. Only miRNA-target interactions showing an MFE of less than − 18.0 were maintained for analysis. We compared these results with the miRNA expression data obtained in 185 animals of our population^31^. After that, we used MiRWalk software^32^ to identify if the ASE genes affected by these aseQTLs were miRNA targets identified by RNAhybrid^80^. For this, we selected the bovine model in the species panel on MiRWalk and searched for genes that were found in the literature as targets for these miRNAs. This list of genes was used for further comparisons with the ASE genes affected by aseQTLs within miRNA binding sites.

## Supplementary Information

Supplementary information.
